# Omental patch-assisted endoscopic closure of the transmural defect after endoscopic removal of a 40-mm gastric gastrointestinal stromal tumor

**DOI:** 10.1055/a-2218-2416

**Published:** 2023-12-21

**Authors:** Renato Medas, Julia L. Gauci, Clarence Kerrison, Francesco Vito Mandarino, Anthony Whitfield, Nicholas G. Burgess, Michael J. Bourke

**Affiliations:** 1 8539 Department of Gastroenterology and Hepatology, Westmead Hospital, Westmead, Australia; 2 285211 Department of Gastroenterology, Centro Hospitalar Universitário de São João, Porto, Portugal; 3 26705 University of Porto Faculty of Medicine, Porto, Portugal; 4 7799 The University of Sydney Sydney Medical School, Sydney, Australia

A 55-year-old woman with a gastric subepithelial lesion (SEL) was referred for treatment. Endoscopic ultrasound (EUS) revealed a 38 × 31-mm hypoechogenic lesion, originating in the muscularis propria, suggestive of a gastrointestinal stromal tumor (GIST). Computed tomography excluded extramural or metastatic disease.

Gastroscopy revealed a 40-mm SEL in the greater curvature of the proximal gastric body ( Fig. 1 **a** ). Exposing endoscopic full-thickness resection (EFTR) by endoscopic submucosal dissection was then performed. After lesion marking and submucosal injection (Gelofusine, indigo carmine, and adrenaline 1:100000) had been performed, the mucosal incision and submucosal dissection were started from the pyloric side of the lesion. After reaching the lesion, a circumferential mucosal incision was completed, followed by tissue traction (clip-and-snare method) to allow better exposure of the submucosal plane. Progressive submucosal dissection was performed around the SEL until the portion attached to the muscularis propria was isolated ( Fig. 1 **b** ). This attachment was then divided at the serosal level to achieve successful en bloc resection.

**Fig. 1:**
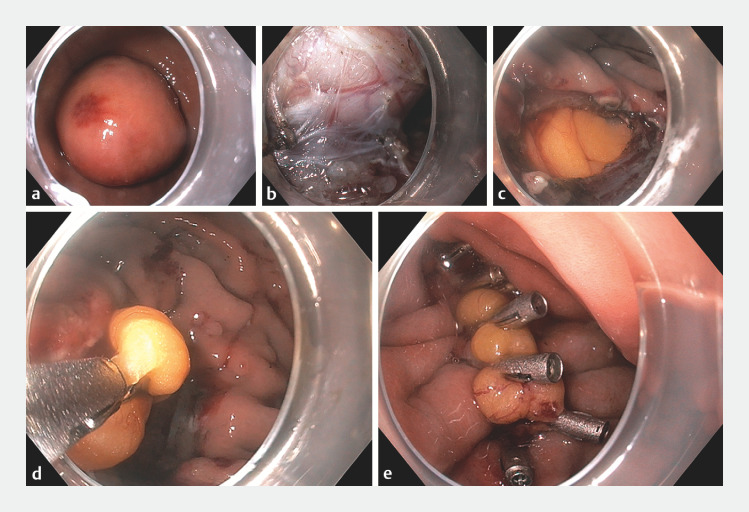
Endoscopic views showing: **a** a 40-mm subepithelial lesion located in the greater curvature of the proximal gastric body; **b** the partially dissected subepithelial lesion, attached only by the muscularis propria on the pyloric side; **c** the resection bed, with a 25-mm transmural defect and omental fat visible underneath; **d** the omentum being grasped with a through-the-scope (TTS) clip and pulled into the gastric cavity to create an omental patch; **e** complete defect closure using the omental patch and seven TTS clips.

As expected, a 25-mm transmural defect was evident ( Fig. 1 **c** ). First, a 20-mm through-the-scope (TTS) clip was placed onto the normal tissue to oppose the defect edges and raise a tissue mound. A TTS clip was then used to pull the omentum into the gastric cavity and create an omental patch ( Fig. 1 **d** ). TTS clips were used to secure the omentum and oppose the defect edges in a zipper fashion ( Fig. 1 **e** ; Video 1 ). The patient was pain-free post-procedure and was discharged 2 days later. No adverse events occurred.

Omental patch-assisted endoscopic closure of a transmural defect, using through-the-scope clips, after endoscopic full-thickness resection of a 40-mm gastric gastrointestinal stromal tumor.Video 1

Endoscopic resection of gastric GISTs is considered a valid and safe alternative to surgery 1 2 . A potential limitation of this technique may be the challenge of closing a wide full-thickness defect. As we present here, one option is the use of an omental patch, which may allow the successful closure of larger defects.

Endoscopy_UCTN_Code_TTT_1AO_2AG

## Notes

Endoscopy E-Videos https://eref.thieme.de/e-videos
*E-Videos* is an open access online section of the journal *Endoscopy* , reporting on interesting cases and new techniques in gastroenterological endoscopy. All papers include a high-quality video and are published with a Creative Commons CC-BY license. Endoscopy E-Videos qualify for HINARI discounts and waivers and eligibility is automatically checked during the submission process. We grant 100% waivers to articles whose corresponding authors are based in Group A countries and 50% waivers to those who are based in Group B countries as classified by Research4Life (see: https://www.research4life.org/access/eligibility/ ). This section has its own submission website at https://mc.manuscriptcentral.com/e-videos .
